# Blood transfusion reactions and risk of acute kidney injury and major adverse kidney events

**DOI:** 10.1007/s40620-023-01859-7

**Published:** 2024-01-29

**Authors:** Fidra Margarita De La Vega-Méndez, Miguel Ibarra Estrada, Esperanza Elizabeth Zuno-Reyes, Carmen Alejandra Gutierrez-Rivera, Ana Elisa Oliva-Martinez, Bladimir Díaz-Villavicencio, Clementina Elizabeth Calderon-Garcia, Jose David González-Barajas, Manuel Arizaga-Nápoles, Fernanda García-Peña, Gael Chávez-Alonso, Adanari López-Rios, Juan Alberto Gomez-Fregoso, Francisco Gonzalo Rodriguez-Garcia, Guillermo Navarro-Blackaller, Ramón Medina-González, Luz Alcantar-Vallin, Guillermo García-García, Gabriela Jazmin Abundis-Mora, Alejandro Martínez Gallardo-González, Jonathan Samuel Chavez-iñiguez

**Affiliations:** 1 Nephrology Service, Hospital Civil de Guadalajara Fray Antonio Alcalde, Hospital 278, Colonia Centro, C.P. 44150 Guadalajara, Jalisco Mexico; 2 University of Guadalajara Health Sciences Center, Guadalajara, Jalisco Mexico; 3 Intensive Care Unit, Hospital Civil of Guadalajara Fray Antonio Alcalde, Guadalajara, Jalisco Mexico; 4 Blood Bank of the Hospital Civil of Guadalajara Fray Antonio Alcalde, Guadalajara, Jalisco Mexico

**Keywords:** Acute kidney injury, Blood transfusions, Major adverse kidney events, Transfusion reactions

## Abstract

**Background:**

Blood transfusion reactions may have a negative impact on organ function. It is unknown whether this association holds true for acute kidney injury (AKI). Therefore, we conducted a cohort study to assess the association between transfusion reactions and the incidence of AKI and major adverse kidney events.

**Methods:**

In this retrospective cohort study, we included patients who received transfusion of blood products during hospitalization at the Hospital Civil of Guadalajara. We analyzed them according to the development of transfusion reactions, and the aim was to assess the association between transfusion reactions and AKI during long-term follow-up.

**Results:**

From 2017 to 2021, 81,635 patients received a blood product transfusion, and 516 were included in our study. The most common transfusion was red blood cell packaging (50.4%), fresh frozen plasma (28.7%) and platelets (20.9%); of the 516 patients, 129 (25%) had transfusion reactions. Patients who had transfusion reactions were older and had more comorbidities. The most common type of transfusion reaction was allergic reaction (70.5%), followed by febrile nonhemolytic reaction (11.6%) and anaphylactoid reaction (8.5%). Most cases were considered mild. Acute kidney injury was more prevalent among those who had transfusion reactions (14.7%) than among those who did not (7.8%), *p* =  < 0.01; those with AKI had a higher frequency of diabetes, vasopressors, and insulin use. Transfusion reactions were independently associated with the development of AKI (RR 2.1, *p* =  < 0.02). Major adverse kidney events were more common in those with transfusion reactions. The mortality rate was similar between subgroups.

**Conclusion:**

In our retrospective cohort of patients who received blood product transfusions, 25% experienced transfusion reactions, and this event was associated with a twofold increase in the probability of developing AKI and some of the major adverse kidney events during long follow-up.

**Graphical abstract:**

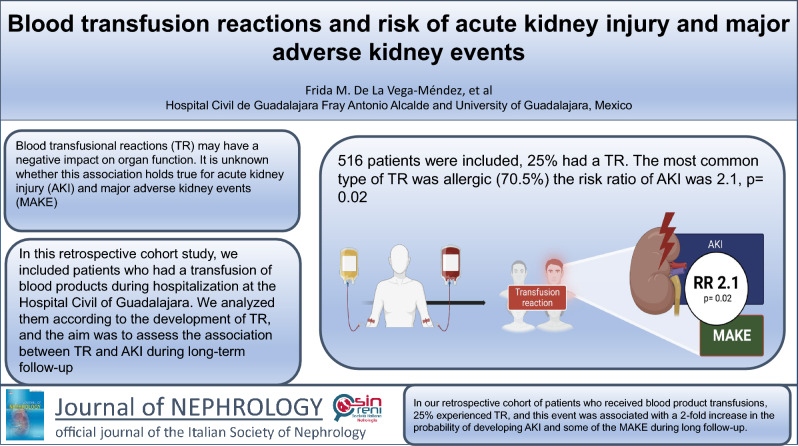

## Introduction

During the hospitalization of critically ill patients, it is common to observe complications such as bleeding [1], severe anemia [2, 3], thrombocytopenia [4], coagulation disorders [5] and hemodynamic needs that require transfusions of blood products. Although blood transfusions have saved millions of lives, it is a procedure that is not free of complications. Transfusion reactions are the most frequent adverse event associated with the administration of blood products, occurring in up to one in 100 transfusions. [6]. When transfusing blood products, an incidence of transfusion reactions of 1.7–4.3 per 100,000 transfusions of red blood cells and plasma has been reported, compared with 62.6 per 100,000 in platelets [7]. These adverse events range from mild, such as generalized discomfort, fever, tachycardia, rash, and hypotension, to severe, such as anaphylactic reactions that could jeopardize the patient’s life [8]. These events are mainly mediated by potent systemic allergic and inflammatory responses [9], which may impact systemic function. These transfusion-associated adverse reactions have been associated with the deterioration of organ function, such as in the lungs, hematological system [10], neurological system with altered alertness or cardiovascular system with shock [11]. The kidney is an organ that could be susceptible to damage during transfusion reactions because it is exposed to high amounts of blood [9, 12]; kidneys have direct contact with inflammatory molecules and can also suffer from abrupt hemodynamic changes that compromise metabolic demands and oxygenation of tubular tissue [13]. However, in a review about transfusion reactions, the Biomedical Excellence for Safer Transfusion (BEST) Collaborative group did not report the incidence of AKI [6]. AKI occurs in up to 23% of critically ill patients, with approximately 10% requiring kidney replacement therapy (KRT), and approximately 50% of patients dying during follow-up [14]. Hence, there may be an association between transfusion reactions and the incidence of AKI and major kidney adverse events in the short and intermediate term. To fill this information gap, we conducted a retrospective cohort study comprising patients who received a blood transfusion during hospitalization and had transfusion reactions to observe whether this event was associated with AKI and major adverse kidney events.

## Materials and methods

### Study design and patient population

This is a retrospective cohort study conducted at the Hospital Civil of Guadalajara Fray Antonio Alcalde, a tertiary referral academic center with 964 beds located in Mexico. All patients included in this study received at least 1 blood product transfusion during hospitalization. We divided the products into red cell concentrate, platelets, cryoprecipitates, fresh frozen plasma, and platelet apheresis. The blood bank closely monitors all blood transfusions by on-site recording data. Transfusion-related reactions were defined as the occurrence of allergic, nonhemolytic febrile, transfusion-associated dyspnea, anaphylactoid reaction, transfusion-associated circulatory overload, and anaphylactic reaction during transfusion. For the AKI event, we chose a 10-day follow-up period after transfusions because most AKI patients start KRT and/or die during this timeframe [15]. Acute kidney injury was diagnosed using the serum creatinine (sCr) KDIGO criteria, and chronic kidney disease (CKD) was defined as an estimated glomerular filtration rate (eGFR) of less than 60 ml/min/1.73 m^2^ for over three months [15]. A major adverse kidney event may result in death, new requirement for dialysis, and worsening of kidney function, defined by *a* ≥ 25% decline in the eGFR from baseline. We chose the major adverse kidney event outcomes due to the recommendation to assess homogeneous results in studies conducted on AKI patients [16]. For a total follow-up, we assessed major adverse kidney events over 30, 90 and 180 days after the index event day (day of the transfusion reactions). All patients included in the study signed informed consent before each transfusion. Transfusion information documents were available for all patients included in the study, as were baseline sCr defined as a pretransfusion sCr value and at least one post-transfusion sCr measurement during hospitalization; those who had sCr in the following months were included in the corresponding major adverse kidney events analyses. Exclusion criteria were AKI patients before transfusion, CKD stage 5, chronic dialysis, hospital stay less than 48 h, kidney transplant, pregnancy, and missing data that would render analysis incomplete.

The study was approved by the Hospital Civil de Guadalajara Fray Antonio Alcalde Institutional Review Board (HCG/FAA/CEI137/23) and was conducted in accordance with the Declaration of Helsinki. Informed consent was waived for the study. The study protocol adhered to the Strengthening the Reporting of Observational Studies in Epidemiology (STROBE) guidelines [17] and the REporting of studies Conducted using Observational Routinely-collected health Data (RECORD) statement [18].

### Data collection

Clinical characteristics, demographic information, and laboratory data were collected prospectively using automated retrieval from the institutional electronic medical record system. Demographic and clinical data (including age, diabetes, hypertension, hypothyroidism, chronic heart failure, CKD grade, cerebrovascular disease, and ischemic heart disease) were collected. Baseline serum creatinine level was defined as the most recent value within a year prior to admission; contributing factors of AKI, such as some nephrotoxic drugs (aminoglycosides, non steroidal anti-inflammatory drugs [NSAIDs], vancomycin and amphotericin B) and shock (administration of vasopressors for a mean arterial pressure < 65), as well as biochemical data such as hemoglobin, platelets, leukocytes, glucose, urea, creatinine, sodium, potassium, chloride, phosphate and calcium, were also recorded. Indications for KRT included fluid overload that was resistant to diuretics, severe hyperkalemia, severe metabolic acidosis, and uremic manifestations, such as encephalopathy, pericarditis, and seizures [15, 19, 20]. The transfusion data were collected prospectively by a physician who then specifically looked for the most common adverse events that might occur during a transfusion of blood products.

### Study outcomes

The primary objective of this study was to determine the association between transfusion reactions and the risk of AKI. The secondary objectives were to investigate the association between transfusion reactions and major adverse kidney events at 30, 90 and 180 days from the index event and to describe the characteristics of patients who developed transfusion reaction-associated AKI and the trajectory of sCr values.

### Statistical analysis

Continuous variables were reported as medians [interquartile range (IQR)], as they were not normally distributed according to the Shapiro‒Wilk test. The Mann‒Whitney test was used for comparisons between groups as appropriate. Categorical variables were expressed as the number of measurements (%) and were compared by the *χ*^2^ test. We used a repeated-measures ANOVA test for comparison of variables over time between groups. Multivariate logistic regression was performed to identify factors associated with AKI development. Calibration was assessed using the Hosmer‒Lemeshow goodness-of-fit test, which was considered adequate if *P* > 0.05. For all tests, *P* values were two-sided, and a value lower than 0.05 was considered statistically significant. We used MedCalc (Ver 22.0.0, Ostend, Belgium) and GraphPad Prism (Ver 10.0.0, Boston, Massachusetts USA) for figures and statistical analyses.

## Results

**Fig. 1:**
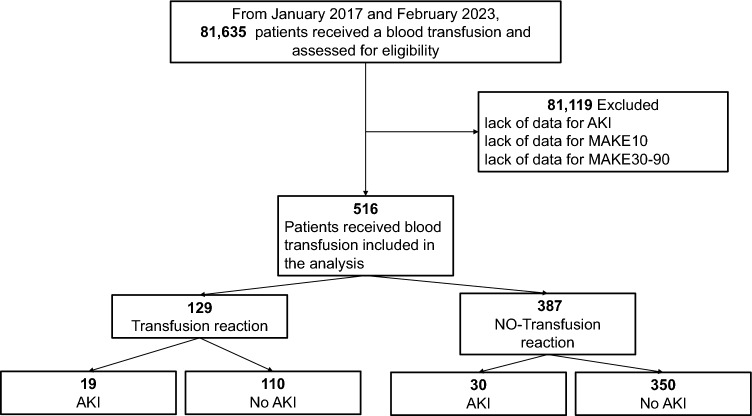
Flow chart of the study

From January 2017 to December 2021, a total of 81,635 patients received a blood transfusion, and 81,119 were excluded. These patients were excluded since there were no available serum creatinine values during follow-up, or data that would allow us to evaluate major adverse kidney events*.* The remaining 516 patients were included in the analysis, of whom 129 (25%) had a transfusion reaction, as shown in the flow chart of Fig. 1. The median age was 43 years (IQR 29–58). The patients in the transfusion reaction group, compared to those who had no reaction, were younger, had fewer comorbidities except cancer, and used fewer antihypertensives. A comparison of baseline characteristics between patients with and without transfusion reactions is shown in Table 1. The transfusion reaction group had lower median arterial pressure and lower platelet count, urea phosphate, and calcium. The most common blood product transfused was red cell concentrates in 260 (50.4%) patients, followed by fresh frozen plasma in 148 (28.7%) and platelets in 108 (20.9%). The most common type of transfusion reaction was allergic (70.5%), followed by febrile nonhemolytic (11.6%), anaphylactoid (8.5%), dyspnea-associated (6.2%), transfusion-associated circulatory overload (1.6%) and anaphylactic (1.6%). The most frequent clinical manifestation was rash in 62.5% of cases, dyspnea in 18% and fever in 13.3%. Regarding severity, most cases were considered mild (82.9%), with 14.7% presenting as moderate and only 2.3% presenting as severe. The length of hospital stay was 10 days (IQR 5–15) in patients with transfusion reactions and 13 days (IQR 8–22) in those without transfusion reactions (*p* < 0.001). Mortality between patients with and without transfusion reactions was similar (7% vs. 6.5%, respectively; *p* = 0.83).

**Table 1:** Baseline and clinical characteristics of transfused patients according to those who developed a transfusion reaction

	TR (*n* = 129)	No TR (*n* = 387)	*p*
Age—years (Q1-Q3)	37 (24–53)	46 (33–58)	< 0.001
Male gender—*n* (%)	66 (51.2)	195 (50.4)	0.87
Body mass index—kg/m^2^ (Q1-Q3)	24.3 (21.7–27.7)	23.3 (21.1–27.0)	0.08
Medical diagnosis—*n* (%)^a^	81 (62.8)	201 (51.9)	0.03
Diabetes—*n* (%)	16 (12.4)	101 (26.1)	0.001
Hypertension—*n* (%)	16 (12.4)	120 (31)	< 0.001
Hypothyroidism—*n* (%)	0	14 (3.6)	0.02
Chronic heart failure—*n* (%)	0	14 (3.6)	0.02
Chronic obstructive pulmonary disease—*n* (%)	2 (1.6)	3 (0.8)	0.60
Chronic kidney disease—*n* (%)	0	0	-
Cerebrovascular disease—*n* (%)	1 (0.8)	1 (0.3)	0.43
Ischemic cardiomyopathy—*n* (%)	3 (2.3)	22 (5.7)	0.15
Cancer—*n* (%)	40 (31)	23 (5.9)	< 0.001
Cirrhosis—*n* (%)	4 (3.1)	13 (3.4)	1.0
Drugs received—*n* (%)			
NSAIDs—*n* (%)	60 (46.5)	215 (55.6)	0.07
Antibiotics—*n* (%)	64 (49.6)	198 (51.2)	0.76
Antihypertensive drugs—*n* (%)	4 (3.1)	72 (18.6)	< 0.001
Diuretics—*n* (%)	3 (2.3)	14 (3.6)	0.58
Vasopressors—*n* (%)	13 (10.1)	22 (5.7)	0.10
Statins—*n* (%)	3 (2.3)	25 (6.5)	0.07
Thromboprophylaxis—*n* (%)	11 (8.5)	9 (2.3)	0.003
Insulin—*n* (%)	22 (17.1)	89 (23)	0.17
Proton pump inhibitors—*n* (%)	96 (74.4)	292 (75.5)	0.81
Blood product—*n* (%)			
RBC—*n* (%)	65 (50.4)	195 (50.4)	1.0
FFP—*n* (%)	37 (28.7)	111 (28.7)	1.0
Platelets—*n* (%)	27 (20.9)	81 (20.9)	1.0
Heart rate—beats/min (Q1-Q3)	88 (79–100)	89 (82–99)	0.25
Respiratory rate—breaths/min (Q1-Q3)	20 (18–20)	19 (18–20)	0.67
Temperature—°C (Q1-Q3)	36.5 (36.0–36.9)	36.7 (36.4–37.0)	0.01
Mean arterial pressure—mmHg (Q1-Q3)	86 (76–90)	91 (83–100)	< 0.001
Hb—g/dL (Q1-Q3)	7.8 (6.4–12.3)	8.2 (6.4–11.6)	0.62
Platelets–cel/µLx10^3^ (Q1-Q3)	166 (75–307)	210 (92–355)	0.01
WBC–cel/µL × 10^3^ (Q1-Q3)	10.1 (7.2–13.5)	10.5 (7.7–13.3)	0.58
Glucose—mg/dL (Q1-Q3)	95 (82–117)	90 (80–133)	0.48
Urea—mg/dL (Q1-Q3)	28.8 (22.0–41.8)	39.0 (32.5–44.0)	< 0.001
Creatinine—mg/dL (Q1-Q3)	0.6 (0.4–0.7)	0.6 (0.5–0.8)	0.55
Na—mEq/L (Q1-Q3)	135 (132–136)	135 (133–137)	0.11
Cl—mEq/L (Q1-Q3)	99 (97–102)	100 (99–105)	< 0.001
K—mEq/L (Q1-Q3)	3.8 (3.5–4.1)	3.8 (3.6–4.0)	0.31
PO_4_—mEq/L (Q1-Q3)	3.6 (3.1–3.9)	3.8 (3.5–4.0)	0.002
Ca—mEq/L (Q1-Q3)	8.4 (8.0–9.0)	8.1 (7.9–8.3)	< 0.001

Medians with interquartile ranges are in parentheses

*Ca*, calcium; *Cl*, chloride; *Hb*, hemoglobin; *K*, potassium; *TR*, transfusion reaction; *NSAIDs*, nonsteroidal anti-inflammatory drugs; *RBC*, red blood cells; *FFP*, fresh frozen plasma; *Na*, sodium; *PO4*, phosphorus; *WBC*, white blood cells

^a^Reference is medical versus surgical main diagnosis

Table 2 shows the subgroups according to the development of AKI.

**Table 2:** Demographic and clinical characteristics of patients who developed AKI

	AKI (*n* = 49)	No AKI (*n* = 467)	*p*
Age– years (Q1-Q3)	47 (27–60)	43 (29–57)	0.26
Body mass index– kg/m^2^ (Q1-Q3)	24.4 (22.0–27.4)	23.5 (21.1–27.2)	0.17
Medical diagnosis—*n* (%)^a^	32 (65.3)	250 (53.5)	0.11
Male gender—*n* (%)	24 (49.0)	231 (49.5)	0.94
Comorbidities—*n* (%)			
Diabetes—*n* (%)	17 (34.7)	100 (21.4)	0.03
Hypertension—*n* (%)	13 (26.5)	123 (26.3)	0.97
Hypothyroidism—*n* (%)	1 (2.0)	13 (2.8)	0.76
Chronic heart failure—*n* (%)	3 (6.1)	11 (2.4)	0.12
Chronic obstructive pulmonary disease—*n* (%)	0	5 (1.1)	0.46
Chronic kidney disease—*n* (%)	0	0	–
Cerebrovascular disease—*n* (%)	1 (2.0)	1 (0.2)	0.18
Ischemic cardiomyopathy—*n* (%)	4 (8.2)	21 (4.5)	0.28
Cancer—*n* (%)	4 8.2)	59 (12.6)	0.49
Cirrhosis—*n* (%)	3 (6.1)	14 (3.0)	0.21
KDIGO 1—* n* (%)	40 (84)	0	–
KDIGO 2—* n* (%)	4.2 (10)	0	–
KDIGO 3—* n* (%)	12.5 (6)	0	–
Drugs received—*n* (%)			
NSAIDs—*n* (%)	24 (49.0)	251 (53.7)	0.52
Antibiotics—*n* (%)	29 (59.2)	233 (49.9)	0.21
Antihypertensive drugs—*n* (%)	7 (14.3)	69 (14.8)	0.92
Diuretics—*n* (%)	2 (4.1)	15 (3.2)	0.74
Vasopressors—*n* (%)	9 (18.4)	26 (5.6)	0.003
Statins—*n* (%)	2 (4.1)	26 (5.6)	1.0
Thromboprophylaxis—*n* (%)	3 (6.1)	17 (3.6)	0.39
Insulin—*n* (%)	20 (40.8)	91 (19.5)	0.001
Proton pump inhibitors—*n* (%)	37 (75.5)	351 (75.2)	0.95
Transfusion reaction—*n* (%)	19 (39)	30 (6)	0.01
Blood product—*n* (%)			
RBC—*n* (%)	30 (61.2)	230 (49.3)	0.11
FFP—*n* (%)	14 (28.6)	134 (28.7)	1.0
Platelets—*n* (%)	5 (10.2)	103 (22.1)	0.06

^a^Reference is medical versus surgical main diagnosis

**Fig. 2:**
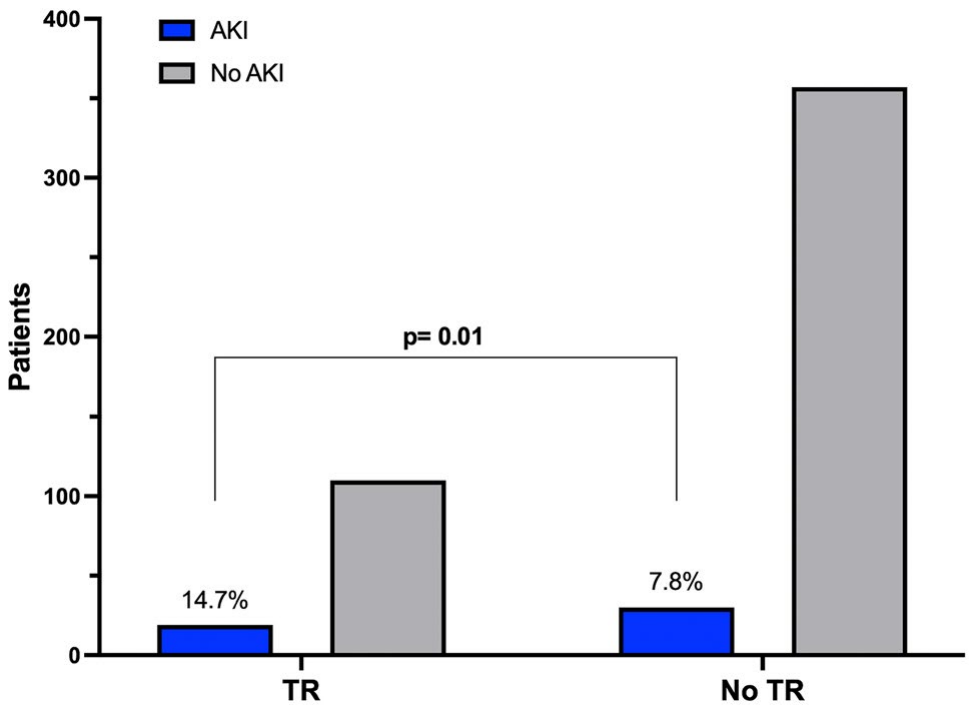
Frequency of AKI in patients who developed transfusion reactions

**Fig. 3:**
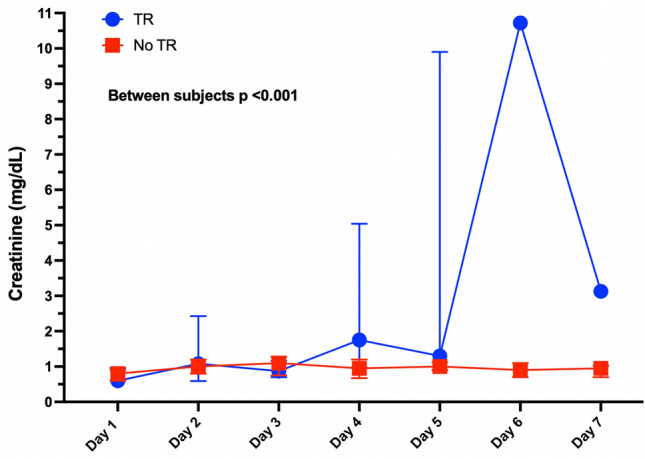
Creatinine trajectory according to whether patients developed transfusion reactions

Acute kidney injury was more prevalent among those who had transfusion reactions (19, 14.7%) than among those who did not (30, 7.8%), *p* = 0.01. (Fig. 2). The vast majority (84%) were KDIGO 1. As expected in patients with AKI, the creatinine levels started to rise after Day 4 and reached significance on Day 6 compared to those with no transfusion reactions, as shown in Fig. 3. The main differences between the patients who received a blood product transfusion and had AKI and those who did not have AKI are presented in Table 2. There were essentially no differences between the groups, except that those in the AKI group had a higher frequency of diabetes, vasopressor use, insulin use, and transfusion reactions (*p* =  < 0.05, for all). Interestingly, there were no differences between the type of blood component transfused and the development of AKI. Management consisted of withholding nephrotoxic drugs (93.7%), adjusting fluid intake (87.5%), antibiotic dose (60.4%), nutritional intake (100%), insulin dose (27.1%), and changing to balanced crystalloids (45.8%). The KDIGO classification of AKI was 1 in 83.3% of cases, 2 in 4.2%, and 3 in 12.5%. In a multivariate analysis with a logistic regression model, transfusion reactions were independently associated with the development of AKI (RR 2.1, *p* = 0.02). A previous diagnosis of diabetes and the use of vasopressors were also independently associated with AKI (Table 3).

**Table 3:** Univariate and multivariate adjusted analysis between variables associated with AKI in patients who received a blood product transfusion

	Univariate analysis			Multivariate analysis		
	AKI (*n* = 49)	No AKI (*n* = 467)	*p*	Adjusted RR	CI95%	*p*
Body mass index– kg/m^2^	24.4 (22.0–27.4)	23.5 (21.1–27.2)	0.17	1.01	0.96–1.06	0.59
Medical diagnosis—*n* (%)^a^	32 (65.3)	250 (53.5)	0.11	1.55	0.78–3.09	0.20
Diabetes	**17 (34.7)**	**100 (21.4)**	**0.03**	**2.04**	**1.02–4.12**	**0.04**
Chronic heart failure	3 (6.1)	11 (2.4)	0.12	1.60	0.37–6.87	0.52
Cerebrovascular disease	1 (2.0)	1 (0.2)	0.18	3.51	0.15–78.9	0.42
Vasopressors	**9 (18.4)**	**26 (5.6)**	**0.003**	**3.49**	**1.40–8.67**	**0.006**
Transfusion reaction—*n* (%)	19 (38.8)	30 (23.6)	0.01	2.10	1.09–4.03	0.02
RBC	30 (61.2)	230 (49.3)	0.11	1.02	0.48–2.16	0.95
Platelets	5 (10.2)	103 (22.1)	0.06	0.42	0.14–1.26	0.12

Goodness-of-fit (Hosmer‒Lemeshow). χ^2^ = 3.95, *P* = 0.86; AUC, 0.71 (0.67–0.75)

^a^Reference is medical versus surgical main diagnosis

Statistically significant results are highlighted in bold

Regarding major adverse kidney events at 30 days, 8 patients (16.7%) had worsening kidney function, 1 (2.1%) needed KRT, and 8 (16.7%) died. At 90 days, 7 patients (14.6%) had worsening kidney function, 3 (6.2%) needed KRT, and 2 (4.2%) died. At 180 days, 5 patients (10.4%) had worsening kidney function, 4 (8.3%) needed KRT, and 2 (4.2%) died.

Regarding the separate outcomes of major adverse kidney events at any time, patients with transfusion reactions were associated with an increased risk of these events. Nine patients (47.4%) had transfusion reactions, and 8 (26.7%) did not show worsening kidney function (*p* = 0.21). Kidney replacement therapy was needed in 4 (21.1%) patients with transfusion reactions and in 0 patients without (*p* = 0.01). The mortality rate was similar between groups (15.8% in transfusion reactions vs. 26.7% without, *p* = 0.49).

## Discussion

In this retrospective cohort, we found that patients who have transfusion reactions have a twofold increased risk of developing AKI during hospitalization, as well as needing additional KRT. To our knowledge, this is the first time that this association has been analyzed.

The occurrence of transfusion reactions could be related to the characteristics of our patients; those who suffered from transfusion reactions had a higher frequency of cancer, lower mean arterial pressure, and a lower number of platelets. It is plausible that these differences conferred some susceptibility for transfusion reactions to occur more frequently, as reported in a previous study [6].

The incidence of transfusion reactions reported in our blood bank is 1.4%. The high incidence of transfusions in our cohort (25%) is much higher than that reported in other studies [6]. We included patients who met the inclusion criteria in the final analysis, which concentrates a population that inherently has more comorbidities and susceptibilities for transfusion reactions. The incidence of AKI in our cohort of patients who received transfusions was close to 10%, a frequency consistent with that presented in other epidemiological studies that are not solely focused on intensive care units [7]. When analyzed according to transfusion reaction status, it was noted that AKI was 6 times more frequent in those who developed transfusion reactions than in those who did not, even after adjustment for multiple confounding variables, yielding a twofold risk.

Half of the transfusions in our cohort were of red blood cells, which is consistent with data from hospitals similar to ours [21].

The risk of transfusion reactions could be an immediate hypersensitivity reaction, a very rare but life-threatening complication. In a US report of transfusion data, there were 71 fatalities among recipients of a blood transfusion, of which 40 were considered directly related to the transfusion, 45% were caused by transfusion-related lung injury, 20% were caused by transfusion-associated circulatory overload, 10% were due to anaphylaxis and 18% were due to other transfusion reactions, but no AKI episodes were mentioned [22]. The risk of IgE-mediated anaphylaxis is estimated at 1 in 20,000 to 1 in 50,000 per unit of blood transfused [8]. The relationship between blood transfusions and the incidence of AKI has been widely studied, especially in the setting of cardiac surgery, where the risk of developing AKI goes hand in hand with the number of transfusions received and is associated with long-term mortality [23–25]. However, these studies did not report whether this phenomenon is associated with transfusion reactions.

In our cohort, we focused on transfusion reactions and their association with AKI. To our knowledge, nothing similar has ever been reported before. We believe that there are arguments that can explain why there is such an association. During transfusion reactions, inflammatory factors such as interleukins [26] and damage molecule-related products (DAMPs) [27] are released into the bloodstream, which filter through the glomerulus, severely affecting the metabolism of tubular cells, altering their function and amplifying tissue damage [28]. Hemolysis also occurs, which affects the kidney parenchyma [11]. Transfusion reactions can induce hypotension with capillary dilation, vascular stasis, and extravasation into the interstitium [12] and thereby decrease the perfusion pressure of the nephron [9], generating an ischemic environment, especially in areas susceptible to hypoxia, such as segments s1, s2, and s3 of the proximal tubule [13]. Hypotension may also occur during transfusion reactions and generate neurohormonal overactivation, decreasing urinary output and sodium excretion [29]. Another mechanism may be fever [30, 31]; in our cohort, the group of patients who underwent transfusion reactions had a 0.2 °C higher body temperature than the group that did not. It has been reported that GFR decreases when body temperature increases by 2 °C [32], and that fever causes direct cellular damage, local effect stimulation of cytokines and inflammatory response, and systemic effects such as gut bacterial translocation [33, 34]. Additionally, management of fever associated with transfusion reactions could have prompted the administration of antipyretics, which are also associated with AKI [35]. Patients who had transfusion reactions had some comorbidities that could be independently associated with the development of AKI, such as cancer, receiving more thromboprophylaxis, a lower mean arterial pressure (86 vs. 91), and a lower number of platelets.

When focusing on patients who had AKI, most of the variables were similar between the groups. However, in the AKI group, there were more patients with diabetes and insulin use, which increased the risk twofold, an expected and widely identified result [36]. In addition, a greater proportion of these patients needed vasopressors, which is historically documented to be associated with AKI [37], but a novel compelling fact was the high presence of transfusion reactions (39% compared to 6% who did not receive vasopressors).

In the entire cohort, major adverse kidney events occurred in 40 patients during the periods of 30, 90 and 180 days. When analyzing the major adverse kidney events individually, we observed no difference in worsening kidney function or death between the patients who developed transfusion reactions compared to those who did not, but an association was found for the start of KRT, which occurred in 21% of transfusion reaction cases compared to 0% of those who did not have a transfusion reaction (*p* = 0.01).

Our findings have clinically relevant implications. When transfusion reactions occur, clinicians could identify the patients with the highest risk of developing AKI, thereby implementing strategies that facilitate the timely diagnosis of this complication, such as measuring sCr more frequently or quantifying urinary output closely. In addition, treatments that potentially attenuate kidney injury, such as switching to less nephrotoxic drugs, strict control of circulating volume or avoiding fluid overload, could be implemented.

Our study has important limitations. As a single-center study, the external validity of our findings is limited. Data concerning the indication for transfusion were lacking. It is possible that an antipyretic such as paracetamol or even an NSAID such as metamizole may have been administered before transfusion reactions to patients with fever, which has been associated with AKI [35]. We were unable to collect data related to the use of NSAIDs in these patients. Our study included only patients from western Mexico, so extrapolation to other health systems or more ethnically diverse societies cannot be made. Our results are only hypothesis-generating; the association between transfusion reactions and AKI does not imply causality and could have had other contributors that, due to the nature of our retrospective cohort, are impossible to rule out. The sample size was not determined.

The main strength of our cohort study lies in our finding of an association between transfusion reactions, AKI and major adverse kidney events even after adjustment for important confounders.

## Conclusions

In our retrospective cohort of patients who received blood product transfusions, we found that 25% experienced transfusion reactions, and this event was associated with a twofold increase in the probability of developing AKI during hospitalization, which, although mild, is associated with major adverse kidney events. Our results may be useful for generating new hypotheses and to pave the way for further research focusing on this association in a larger population with a more robust study design.

## Data Availability

The files and data are in the physical and electronic archive of the Civil Hospital of Guadalajara Fray Antonio Alcalde and can be requested to the corresponding author. All data generated or analyzed during this study are included in this article. Further inquiries can be directed to the corresponding author.
